# Family Club Denmark #strongertogether - a volunteer intervention for disadvantaged families: study protocol for a quasi-experimental trial

**DOI:** 10.1186/s40359-020-00426-0

**Published:** 2020-06-01

**Authors:** Maiken Pontoppidan, Mette Thorsager, Arendse Tange Larsen, Mette Friis-Hansen

**Affiliations:** VIVE – The Danish Centre for Social Science Research, Herluf Trolles Gade 11, 5200 Copenhagen, Denmark

**Keywords:** Volunteer, Third sector, Parenting, Family, Support, Intervention, Effectiveness

## Abstract

**Background:**

Need-oriented family support programs are examples of voluntary-based interventions increasingly recognized by the public sector as an important contribution to health and social care provision. Voluntary interventions are attractive because of their focus on activism, inclusion, and participation, but also their low cost and easy accessibility. There is an increasing demand for documentation of the effectiveness of family support programs. Methodologically sound studies are, however, limited and findings are generally inconsistent. This trial aims to assess the effectiveness of the volunteer-based intervention Family Club Denmark on parental stress, mental health, development and well-being of parents and children and to get insight into the experiences of both volunteers and families.

**Methods:**

This is a prospective quasi-experimental trial with two conditions: (1) intervention group participating in Family Club Denmark and (2) wait-list control group. Participants are families with children aged 2–12 years who wish to participate in the program. Participants are allocated to intervention primarily after a first-come-first-serve principle, and further families will join the wait-list and be offered participation after around 6 months. Quantitative data are collected through web surveys at three time-points: at baseline, post-intervention (6 months after baseline), and follow-up (12 months after baseline). The primary outcome is mental health. Secondary outcomes include parenting behavior, parenting stress, self-efficacy and self-worth, family routines and child well-being. Qualitative data are collected through observations, focus groups, and interviews.

**Discussion:**

This trial is among the first experimental studies of a group-based third sector need-oriented family support program offered to a wide array of families. The trial will provide important knowledge on the effectiveness of a volunteer-based family intervention on important outcomes such as mental health, self-efficacy, family routines. Furthermore, the trial will provide knowledge on volunteer, parent, and child experiences with participating in the intervention and knowledge on how to conduct experimental trails in a complex volunteer environment.

**Trial registration:**

ClinicalTrials.govNCT03657888 (registered 29.08.2018).

## Background

Civil society is gaining increased attention and recognition as a significant partner in the welfare state. Membership in democratic associations is considered a core element of Nordic welfare and has ensured democratic influence in the community and society as a whole. There is a large degree of cooperation between the state and civil society organizations, and voluntary organizations serve as intermediate institutions between the citizens and the state [1]. In Denmark, associations with member volunteers dominate the tradition and there is a high degree of participation measured per resident in democratic organizational models in local, regional and national formats [1–3]. Voluntary activities arising from civil society are often referred to as ‘the third sector’, as it is delimited from both the private and the public sector. These activities are – due to their innovative solutions to problems and their democratic structures - increasingly regarded as important contributions to solving complex problems such as loneliness, social problems and health problems [4–7]. In Denmark, for instance, civic engagement in health and social care has received awareness and recognition from the Danish Health Authority and the National Board of Social Services) [8–11] for their valuable contribution. The public sector finds the opportunity of drawing on voluntary efforts in the delivery of welfare services appealing because it offers a (partial) solution to scarce public resources and has the potential to cater to new needs and demands in society [4–6]. Hence, voluntary services are intuitively considered valuable; especially in cases where costly professional work is shifted towards non-professional, inexpensive support.

Civil society organizations work with a wide range of different purposes and characteristics, including development from collective to individual engagement, from member-based to program-based efforts, and from institutionalized to self-organized activities. Activities that all coexist with the traditional association- and membership-based civil society organizations [12]. The nature of voluntary activities has evolved during recent decades and volunteerism has recently been subject to increased marketization involving increased demand for and focus on performance, impact, and documentation [13]. This focus can potentially conflict with the democratic and inclusive nature of the civil society [14–16]. Existing literature on the impact of voluntary interventions within the health and social care represents different approaches and methodologies. However, methodologically sound studies such as randomized controlled trials (RCTs) and well-designed cohort studies are limited [17]. Without such evidence, no firm conclusions can be drawn about the impact of volunteer interventions [17]. The current literature covers a broad spectrum of interventions [7, 18–36] ranging from elderly and end-of-life voluntary care [6, 37, 38], voluntary care for left-behind children [22], programs targeting struggling readers [24], prevention and early detection of cancer [39] to need-oriented family support programs [25–28, 32, 33, 40].

The current study represents the latter. The intervention examined in this study is Family Club Denmark (FCD). FCD is a voluntary program targeting vulnerable families by creating communities between families with different social backgrounds supported by one voluntary leader and two-to-five further volunteers. FCD is a family support program aiming to increase the network for both children and parents in vulnerable families and promote positive parenting such as behavior, competence, development and family functioning.

The literature on the effectiveness of volunteer family support programs finds mixed results due to differences in programs (e.g targets and timing), expected outcomes and research design. Kelleher & Johnson evaluated the Cottage Community Care Pilot Project using a descriptive comparative design and concluded that the intervention group experienced greater improvements in seven aspects of family functioning compared to the control group. However, the improvements were only statistically significant for two aspects [25]. Caring in Chaos (CiC) is a parenting intervention targeting families of children with parental concerns about ADHD. In an RCT the intervention group comprising 80 families receiving CiC was compared to a wait-list condition. The trial suggested improved parental behavior, sense of competence, parental stress and depressive symptoms as well as child function at post-treatment. At 4 month’s follow-up, most of these effects were sustained [40]. Gardner et al. carried out an RCT examining the effectiveness of a parenting intervention for reducing child conduct problems.0 76 children aged 2–9 years were randomized to either receiving a video-based 14-week program or to a wait-list control. Comparing the two groups after 18 months indicated significant improvements in outcomes such as observed child negative behavior, child independent play and parents’ sense of competence as well as observed and self-reported positive parenting but with no change in maternal depression [41]. Another example of a family support program is Home-Start which was initiated in the UK in 1973 but has now spread to 22 countries [42]. Home-Start offers volunteer support to vulnerable families with at least one child below 5 years of age [43]. Gentry et al. examined the effectiveness of Home-Start Suffolk (UK) using a mixed-methods design and concluded that stakeholders perceived the program as successfully supporting families in need of additional help. However, poor quality of administrative data hampered a quantitative analysis of effectiveness [4]. Other evaluations of the Home-Start initiative include quasi-experimental studies and uncontrolled studies that have found somewhat mixed results. In a quasi-experimental study, Asscher et al. found positive effects of Home-Start on maternal competence, mixed effects on parenting behavior and no effects on maternal depressive moods and child behavior at the end of the intervention (approx. Six months) [44]. Results were based on self-reported and observational data. Two additional quasi-experimental studies with longer follow-up showed the greatest improvement in parental well-being, behavior, and competence during the intervention, and these changes were sustained until 10 years after the intervention [26, 45]. Barnes et al. evaluated the impact of Home-Start on maternal depression after labor in an uncontrolled randomized study, and found no reduction in the risk of depression among new mothers [27]. This finding is supported by a quasi-experimental study that demonstrated perceived improvements in maternal well-being but failed to demonstrate statistically significant effects on several indicators of maternal well-being including parental stress, maternal depression and maternal self-esteem between the groups compared [43]. In addition to providing inconsistent results, the literature is challenged by shortcomings such as high and potentially selective attrition rates [26, 45], small sample sizes [25–27, 41, 44–46], lack of randomization [26, 44–46], no comparison group [4] and no complete blinding of the observer [44].

Generally, methodologically sound studies on the impact of volunteer family support programs are limited, and findings are generally inconsistent due to differences in study methodologies, outcomes examined and characteristics of the programs investigated. Results of meta-analyses and systematic reviews support this by showing varying effect sizes and directions of effects depending on included studies and outcomes of interest [47–49]. Despite inconsistent results, family support programs carried out by volunteers are still continuously being developed and implemented. However, there is a need for ongoing systematic and rigorous evaluations to document the effectiveness of these interventions. The current study is a prospective quasi-experimental trial contributing to this evidence-base. The trial aims to assess the effectiveness of the volunteer intervention Family Club Denmark (FCD) on parent stress, mental health, development, and well-being of parents and children. We will also examine the implementation of FCD concept across organizations, the experience of volunteering within FCD, and the experiences of the participants. The hypothesis is that families in the FCD group will improve more than the control group on all outcomes.

## Methods and design

The study is a prospective quasi-experimental trial with two conditions: (1) intervention group participating in Family Club Denmark and (2) wait-list control group. The trial aims to examine the effectiveness of Family Club Denmark. The primary outcome of the study is mental well-being and secondary outcomes comprise parenting behavior, parenting stress, self-efficacy and self-worth, family routines, and child well-being. Participants will be allocated to the intervention group primarily after a first-come-first-serve principle. When the maximum number of participants in a specific family club is reached, any further families will join the wait-list and be offered participation after 6 months if a family club exists in the area they live in. The control group will also include families who sign up for a family club in an area where a family club does not yet exist. The study takes place in all five different regions of Denmark. Recruitment commenced in September 2018 and is planned to close in early 2020. We will recruit participants from family clubs starting in fall 2018, spring and fall 2019, and spring 2020. The FCD project is a collaboration between the three third sector partner organizations; KFUMs Sociale Arbejde, FDF, and KFUM Spejderne.

**Fig. 1:**
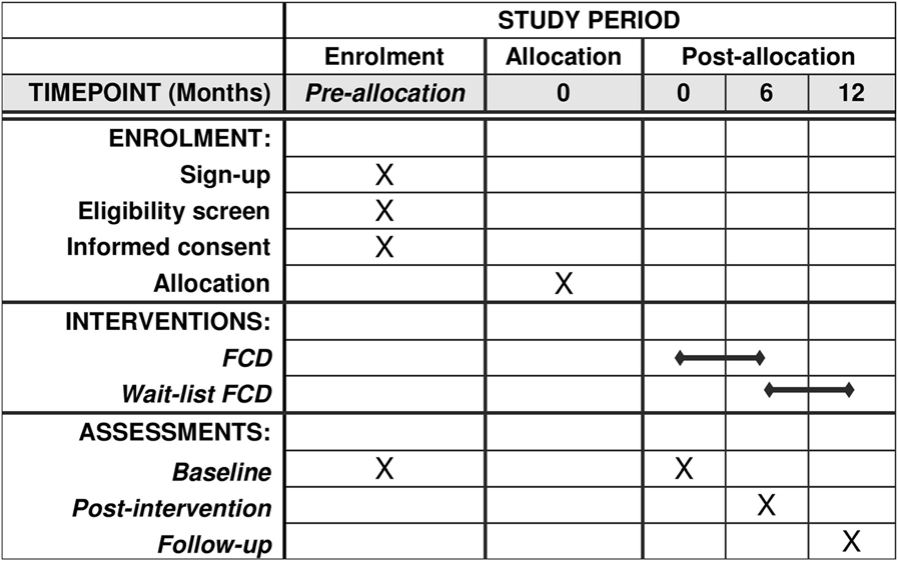
Schedule of enrolment, interventions, and assessments

Participants will receive written information on the project and will give electronic written consent to participate in the trial. Ethical approval has been obtained from the internal review board at VIVE. According to Danish law, the project does not need to obtain approval from The Danish National Committee on Health Research Ethics. The protocol conforms to the Standard Protocol Items: Recommendations for Interventional Trials (SPIRIT) guidelines (Fig. 1). The final reports of the trial will be written following the Consolidated Standards of Reporting Trials (CONSORT) statement.

### Participants

Participants are vulnerable and non-vulnerable families with children aged 2–12 years who wish to participate in FCD. Most vulnerable families will be characterized by at least one of the following characteristics: being a single parent, having low income, having a small or no network, experiencing a low level of support, experiencing loneliness or lack of contact with other adults, having difficulty creating relationships, having physical or mental health problems, or having a hard time making ends meet during the week. To be included in the trial families must have at least one child 2–12 years old and sign up for FCD. Families are excluded if they are not able to fill out questionnaires in Danish. The vulnerability classification of families is based on labor market status, and the motivation for participation in FCD stated at sign up. Families are classified as vulnerable if at least one parent is outside the labor force (for reasons other than enrollment in education) or unemployed and/or the motivation for participation in at least one of following: Our daily life is very stressful, we feel lonely, we have many conflicts in our daily life, we struggle as a family, we find it hard to become part of new social networks.

### Recruitment

We aim to recruit 200 vulnerable families – 80 to wait-list and 120 to FCD. Participants are recruited through the FCD website, by Facebook adds, by municipal social workers, by local social housing employees, and by direct contact with the local FCD volunteers. The three partner organizations have employed project coordinators to find venues, recruit volunteers, recruit families, handle the website and sign-up, and support volunteers. In most cases, the recruitment process is as follows: (1) A family applies for a specific family club through the website and gives consent for VIVE to contact the family; (2) A project coordinator creates a list with participants; (3) The project coordinator allocates participants to intervention or wait-list control; (4) The project coordinator sends contact information and allocation condition to VIVE; (5) The project coordinator informs VIVE about the starting dates of the family clubs; (6) VIVE sends the first questionnaire (including electronic consent to participate) by text-message and e-mail; and (6) The project coordinator informs participants about allocation. Most families are recruited through the FCD website but some are recruited directly at the local site e.g. through an existing scout unit or the local social housing office. In some cases, families will sign up after the FCD has started.

### Allocation

**Fig.2:**
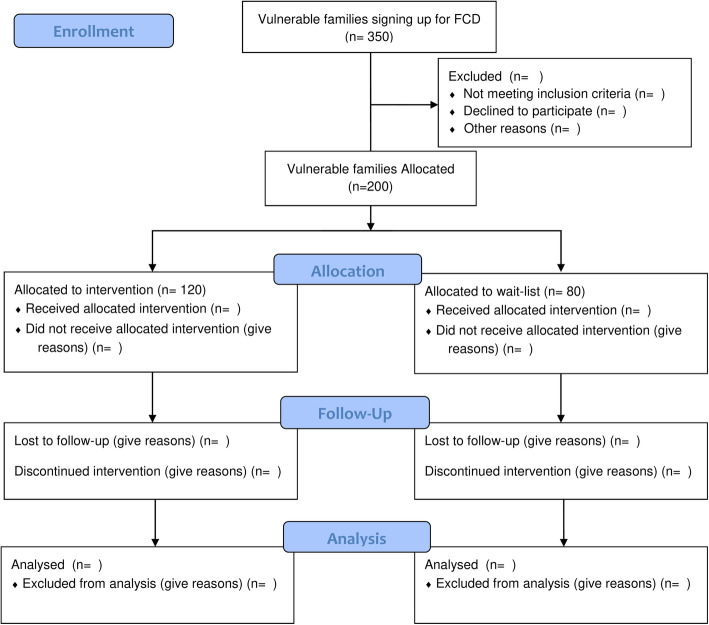
Study flow chart

Families are allocated to a family club or a waitlist based on a first-come-first-serve principle conditional on their vulnerability classification. Around nine families are allocated to one family club and any extra families will be offered to be on a wait-list for the next round or when another club starts in the area at a later timepoint. The coordinators strive to allocate two or three non-vulnerable families to each family group to make sure that non-vulnerable families are present in all clubs. The coordinators also make sure that families with sufficient Danish language skills are present in each club making it possible for families that do not have Danish as their first language to improve their Danish skills. The discretionary allocation of families, carried out by the coordinators, is made without further regards to family characteristics and thus can be considered as good as random conditional on vulnerability status and Danish language skills. Families that sign up for FCD but live in an area where there is currently no FCD are included in the wait-list control group. Families who have been assigned to FCD are included in the wait-list control group if the start of the FCD is delayed by at least 4 months. Families who have been assigned to FCD but are not able to participate, e.g. because they have other obligations on the assigned day, and families who do not show up for FCD are included in the wait-list control group. The flowchart is presented in Fig. 2.

The first-come-first-served principle potentially introduces selection issues if first responders differ from later attendees by e.g. being more enthusiastic. The recruitment strategy, involving the use of several channels that target potential families in very different approaches, can to some extent mitigate the selective timing of sign up. The mix of personal recruitment by municipal social workers or local social housing employees with the online recruitment process can introduce some randomness in who signs up when. Also, first responders can be assigned to the control group for other reasons such as no club in their local area, or the family could not participate on the day that the club was offered. We expect that most families are allocated to the control group because they sign up for an FCD in an area where there is not yet any FCD. Therefore, we expect that the potential selection issues introduced by the first-come-first-served principle are minimized.

### Intervention and training

The FCD concept was developed by the project partners in the developmental part of the project in 2017. The principles are described in a concept book and a practical guide. Theoretically, FCD is based on social learning theory, neuroscience, and positive psychology. The focus is on proving positive experiences for families and building relationships. Volunteers should meet the families with a positive attitude and show them that they each are important contributors to the family club. The materials also recommend that volunteers use praise and apply predictability and routines in the family club. The volunteers should create a program for each session and present it to the families. The program should include the following elements: welcome, activity, dinner, goodbye.

A family club is run by a volunteer team comprised of a leader and two-to-five further volunteers. Each club consists of up to nine families who meet every second week for 6 months (12 sessions). The club runs for 6 months at a time to ensure enough time for relationships to grow and to create a predictable structure around the club. In the first months, the focus is getting to know each other whereas there is more focus on building both the internal and external networks in the last part of the intervention. The aim is that an FCD will run for consecutive periods but families and volunteers sign up for 6 months at a time. If participants wish to stay in the FCD for another 6 months after finishing the first 6 months they can. Although each club includes both vulnerable and non-vulnerable families all families must participate on equal grounds.

The FCD is based on the following four principles: (1) Meal community; (2) Play, learning, and togetherness; (3) Support and advice; and (4) Bridging to the civil society and the public sector. Each meeting in the FCD must include the first two principles, i.e. meal and play. The 12 sessions center around seven value posters that can be put on the wall in the room. The seven values are: (1) Fun with smiles; (2) Together but not in line; (3) The time is now; (4) Notice and say thank you; (5) More than me; (6) Courage to dare; and (7) Taste the world. The seven values are explained in the FCD concept book and activities are linked to the seven values.

Most volunteers will participate in a one-day training before the club starts. The training is based on the four principles and seven value posters but also provides volunteers with guidance on e.g. body language, teamwork, and meeting facilitation. Each training includes a presentation by an external presenter on relevant aspects from social learning theory, neuroscience, or positive psychology. For experienced volunteers, the training session consists of more advanced training and team support. All volunteers receive a print of the FCD concept book explaining the core principles of the FCD and with suggestions for activities and games that can be used in the club. They also receive a guidebook with practical information on issues such as teamwork, what is expected of a volunteer, club economy, fundraising, confidentiality, and insurance.

### Wait-list control group

Families who are on the wait-list will be offered participation in FCD after approximately 6 months. We do not expect all wait-list families to participate in FCD in the second period. Some areas will still not offer an FCD at this time and we also expect that some families may not be interested in participating at this time point. Therefore, we will have both families who receive FCD in the second period and families who never receive FCD in the control group. Families in the wait-list control may participate in other volunteer projects.

### Outcomes

Data are collected through web surveys at three time-points: baseline, post-intervention (6 months after baseline), and follow-up (12 months after baseline). Participants receive an e-mail with a direct link to the questionnaire. Reminders are sent by text message and e-mail. If the families need help to fill out the questionnaire a student will call them. Families receive a small gift at each of the three data collections. At baseline, they receive a cookbook for children, at post-intervention, they receive a 150 DKK (~ 20 EUR) electronic gift card, and at follow-up, they receive a 100 DKK (~ 15 EUR) electronic gift card. When signing up the families are informed that they will receive a small gift after completing each questionnaire. Data are collected in a secured online survey database. The trial statistician and the principal investigator will have access to the full dataset.

### Measures

Table 1 shows the timing of the administration of measures. Socio-demographic characteristics assessed at T1-T3 include the age of the parent, education, occupation, ethnicity, number of children, child age, household status, housing situation, and household economy.

**Table 1:** Timing of outcomes – parents

		T1	T2	T3
**Parent measures**				
Background	Age, gender, language, education	√	√	√
Family	Partner, children	√	√	√
Mental health	Warwick-Edinburgh Mental Wellbeing Scale	√	√	√
Self-efficacy	From general self-efficacy scale	√	√	√
Parental stress	Parental Stress Scale	√	√	√
Family conflicts	Partner, child	√	√	√
Family life	Leisure activities	√	√	√
Family routines	Mealtime, duties, bedtime, homework	√	√	√
Network	Loneliness, practical help, confidants	√	√	√
Parenting competences	From Parent Behavior Inventory	√	√	√
Play	Play with children	√	√	√
Screen time	Parent	√	√	√
Family budget	Worries, budget	√	√	√
Satisfaction	Participation, network		√	
**Child measures**				
Well-being child (< 8)	Well-being	√	√	√
Well-being child (≥8)	Kidscreen 10	√	√	√
Network	Friends	√	√	√
Screen time	Mobile phone, computer	√	√	√
Learning activities	Reading, talking	√	√	√

#### Primary outcome

The primary outcome of the study is the 7-item **Short Warwick-Edinburgh Mental Well-being Scale (SWEMWBS)**. This measure is chosen because it is a relatively general measure of well-being in adults. The SWEMWBS has recently been validated in a Danish sample [50]. A total score is calculated by summing the 7 items and converting the raw score according to a published conversion table. Score range 7–35 for both raw and converted scores. A high score is a better outcome [51]. Cronbach’s alpha is 0.85 on a subsample of the included families.

#### Secondary outcomes

The secondary outcomes include the following measures. It was important to both covers a relatively broad area of outcomes that we believe potentially can be impacted by the intervention but also keep the questionnaire relatively short. Therefore, we have shortened some of the included measures.

**Parent Behavior Inventory (PBI)** is a 20-item measure of parenting behavior of parents of early school-aged children. The PBI includes two subscales with high internal consistency: Supportive/engaged (α = .83) and hostile/coercive (α = .81). The PBI has adequate test-retest reliability (r = .69–.74) in an ethnically diverse sample of mothers. To reduce the total number of items we include 10 items – 5 from each of the two subscales; items 6, 10, 11, 12, 14 for Supportive/Engaged, and items 5, 9, 15, 17, 20 for Hostile/Coercive. The score range is 0–25 for each subscale. A high score is better for Supportive/Engaged, a low score is better for Hostile/Coercive [52]. Cronbach’s alpha is 0.66 for the supportive/engaged subscale and 0.55 for the hostile/coercive subscale in a subsample of the included families.

**The Parenting Stress Scale (PSS)** is an 18-item measure of parenting stress that is rated on a five-point scale (Strongly disagree, Disagree, Undecided, Agree, Strongly Agree). We use the revised Danish version with 16 items where the original items 2 and 11 are left out. The Danish version consists of two subscales: Parental Stress (9 items) and Lack of Parental Satisfaction (7 items). Responses are reversed for the lack of parental satisfaction items and all responses for all items are dichotomized (0–1) before scoring. Total score range 0–9 (Parental Stress subscale) and 0–7 (Lack of Parental Stress subscale). A low score is better for both subscales [53]. Cronbach’s alpha is 0.76 for the parental stress subscale and 0.82 for the lack of parental stress scale in a subsample of the included families.

**The general self-efficacy scale (GSE)** is a 10-item measure of optimistic self-beliefs to cope with a variety of difficult demands in life. To reduce the total number of items we include 3 items (8, 9, and 10). The score range is 3–12 and a high score is better. Cronbach’s alpha for the 3-item version is 0.87 in a subsample of the included families.

**Self-worth** We use 3 items from the HBSC project to measure self-worth. The 3 items are inspired by Rosenberg’s concept of self-esteem. Items are scored from 1 (completely disagree) to 5 (completely agree) and summed. A high score is better [54]. Cronbach’s alpha for the 3-item version is 0.87 in a subsample of the included families.

**Family routine** We use 5 items (1,4,9,11,21) from the Child Routine Inventory (CRI - 39 item version). Item 1,3,4, and 5 are from the Daily Living Routines subscale and item 2 is from the Household Responsibilities subscale. Inspired by the CRI we developed 5 extra items on family routines around mealtimes and language. Items are scored from 0 (never) to 4 (always). The high score is better. Cronbach’s alpha is 0.69 for the daily routines subscale, 0.56 for the family life subscale and 0.49 for the meals subscale in a subsample of the included families.

**Kidscreen-10** is a 10-item measure of child well-being (health-related quality of health). Items are scored from 1 (never) to 5 (always) except for items 1 and 9 (reverse). Items 1 and 2 explore the level of the child’s/adolescent’s physical activity, energy and fitness. Items 3 and 4 covers how much the child/adolescent experiences depressive moods and emotions and stressful feelings. Items 5 and 6 ask about the child’s opportunities to structure and enjoy his/her social and leisure time and participation in social activities. Item 7 explores the quality of the interaction between child/adolescent and parent or carer and the child’s/adolescent’s feelings toward their parents/carers. Item 8 examines the nature of the child’s/adolescent’s relationships with other children/adolescents. Finally, items 9 and 10 explore the child’s/adolescent’s perception of his/ her cognitive capacity and satisfaction with school performance. A higher score is better [55]. Cronbach’s alpha is 0.85 in a subsample of the included families. Kidscreen-10 is used with families where the target child is 8 years old or more. Parents with younger children will receive questions about child well-being from the questionnaire to 2–6-year-old children in the project BørnUngeLiv (boernungeliv.dk). Cronbach’s alpha is 0.64 in a subsample of the included families.

### Volunteers

We invite volunteers in the project to fill out two questionnaires: the first one immediately after the training session and the second one around 4 months after training. The first questionnaire assesses satisfaction with the training session. The second questionnaire includes questions on gender, age, family status, education, employment, motivation for becoming a volunteer, collaboration with the project coordinators, collaboration with other organizations, the collaboration between the volunteers, and characteristics of the family club such as location, number of sessions, and activities. The volunteers also fill out a web-based questionnaire after each session providing information on the date, the number of participants, which value poster and activities are used, what the meal was, and if any guests participated.

### Statistical analysis

We analyze the primary and secondary outcomes at post-intervention (T2) and follow-up (T3). We use linear regression for normally distributed outcomes and apply robust standard errors to correct for the correlation between observations participating in the same FCD group. In the case of heavily skewed data, we use non-parametric rank-based tests. To check whether the first-come-first-served principle or the allocation of vulnerable families conducted by the volunteers introduce imbalances in the allocation to FCD and the waitlist control, we test for statistical differences in observable characteristics of the two groups using t-tests. In the analysis of the effectiveness of FCD, the hypothesis tested for each outcome is that the FCD group will score better than the wait-list control group. We test this hypothesis with a binary variable indicating the intervention group. We include variables measured at baseline with indications of imbalances (*p* < 0.1) as control variables and apply two-sided tests with 0.05 significance levels throughout. As all outcomes represent a different aspect of the expected outcome of the intervention we do not consider multiple testing issues. We analyze primary and secondary endpoints according to an intention-to-treat (ITT) principle and handle missing data with multiple imputations. Parametric imputation models are preferred, but in case of severe non-normality or convergence issues, we use predictive mean matching or other non-parametric options. We include the 12-month outcome data for exploratory analyses but can also be used as tools in secondary analyses of missingness. Exploratory analysis of data from all three measurements (T1 - baseline, T2 - 6 months and T3–12 months) will be carried out using mixed-effect regression and possibly latent growth curve analysis. The purpose is to assess the longer-term effects of the intervention and to understand how the psychosocial characteristics of the families involved develop over time. In addition to the primary analysis, we will perform subgroup analyses to examine potential differences between subsets of participants. Hence, we analyze subgroups according to the following characteristics: vulnerability (vulnerable or non-vulnerable families); family composition (single parents or cohabiting parents); the age of target child (< 8 years old or ≥ 8 years old); partner organization (KFUMs Sociale Arbejde, FDF, or KFUM Spejderne); the number of volunteers in the FCD; and the number of sessions attended (dose).

### Blinding

It is not possible to blind participants or volunteers, as everyone will know if they are participating in FCD or are on a wait-list. The data analyst will be blinded to group status.

### Power considerations

We carried out a power analysis in the design phase to assess the statistical power for testing the main hypothesis. As this study is among the first effectiveness studies of a volunteer intervention there is a lack of previous research to guide the power analysis. Two reviews examine the effect of home visiting interventions (including both professional and paraprofessional providers). Nievar et al. found an average effect size of 0.37 [0.21:0.53] on maternal behavior across 60 studies of home-visiting for at-risk families [56]. The average effect size did not differ significantly between studies with professional and paraprofessional home visitors. Filene et al. found an average effect size of 0.23 [0.13:0.33] on parent behaviors and skills [49]. Two randomized studies examine the effects of volunteer parent training programs for parents with children with ADHD or behavior problems. Chacko & Scavenius report effect sizes post-intervention of 0.3 for parental stress, 0.46 for parenting behavior, and 0.65 for parenting competence [40]. Gardner et al. report effect sizes post-intervention of 0.40 for parenting confidence and 0.65 for parenting skills. None of these studies, however, report results on parental well-being.

We expect controls to constitute approximately 80 out of 200 families. For normally distributed outcomes, a sample of 200 participants (80 control and 120 intervention) would yield a statistical power of approx. 80% with a type 1 error rate of 0.05 for an effect size of 0.35 standardized mean difference (SMD) and a correlation between baseline and post-intervention measures of 0.50.

## Qualitative methods

In addition to the quantitative measures, we will supply with qualitative techniques including observations, focus group interviews and individual interviews. The qualitative parts aim to capture information on the feasibility and acceptability of the FCD and to provide in-depth knowledge of the experiences, interactions, roles, methods, activities, relationships, and perceived outcomes of both volunteers and families in the FCD. We will visit 14 different FCDs at three-time points and conduct observations, focus group interviews and individual interviews with participating parents and children who volunteer to be interviewed. The 14 family clubs are selected to ensure a distribution of characteristics such as urban/rural location, partner organization, single parents/families, so make sure that we gain in-depth knowledge of the variation in FCD and how the family clubs are being implemented in different settings. The qualitative study also includes the organizational framework of the collaboration between the three civil society organizations. This includes how the framing of the FCD affects the interactions and outcomes of the participants. We will conduct interviews with the steering group, the project coordinators, and volunteers.

Specifically, we will investigate the following domains: How family networks develop over time; How relationships between the adults in FCD develop and what it means for the families to participate; How the relationships develop between the children and between the children and the adults and what significance children and adults attribute to the relationship; What roles the volunteers take, what importance they attribute to the families and how and why they are involved (motivated and demotivated); How the volunteers facilitate the family club and what significance it has for the families; What personal benefits parents and children experience from participating in the family club: Whether parents and children feel like equal participants and contributors to the FCD. We will examine changes in these domains during the intervention period and explore the attributions of change. Also, we will perform social mappings of parent networks and conduct group discussions with staff on their perspectives and experiences of using the FCD. The themes guiding our analysis will be drawn from the objectives of the trial but also the data, should additional areas of interest emerge during interviews and discussions.

Senior analysts with extensive experience in carrying out qualitative research with vulnerable groups will collect the qualitative data.

## Discussion

This study is a prospective quasi-experimental trial assessing the effectiveness of FCD on parent stress, mental health, development and well-being of parents and children. The study will contribute to the evidence-base of the effectiveness of need-oriented family support programs.

Compared to other research fields, the tradition of rigorous evaluation of interventions’ impact within civil society is rather weak. The voluntary nature of such interventions is an inherent challenge when evaluating impact. Thus, this is a challenge that this study shares with similar studies examining the impact or effectiveness of other voluntary interventions. Voluntary engagement is difficult to control and although the concept is manualized and volunteers are offered a one-day training the intervention highly depends on the engagement and personal qualities of the volunteers. It is not possible and would not be fruitful to demand the volunteers to act strictly according to a manual. Consequently, the study is conducted in a very flexible and accommodating way. The practical execution is streamlined as much as possible but with a high degree of respect for local needs and differences. Therefore, family clubs will wary in terms of what and how they practically carry out the activities. Moreover, family clubs take place in settings with different characteristics such as the degree of ethnic diversity, public housing, and family income. This is likely to cause variation in delivery and receipt of the FCD activities. The study does provide some insight into these differences in the content of the different FCD’s, and the different competencies of the volunteers. The degree of variation may though complicate the identification of effective components of FCD, including characteristics of both volunteers and participants that contribute to FCD being more or less successful.

Other potential limitations relate to methodological as well as practical challenges. A practical challenge concerns the probability of attrition among participants allocated to the control group; if FCD is perceived as an attractive offer, being assigned to the wait-list with the prospect of being offered the intervention in six months might be difficult to accept. Because we are not able to apply strict random allocation comparability between the intervention group and control group cannot be guaranteed and conclusions about causality are expected to be tentative rather than definitive. On the other hand, the study is conducted in a naturalistic setting, which can be regarded as a strength to represent the setting in which FCD is applied.

## Trial status

Protocol version 1 was dated September 4th, 2018 and the second version dated May 1st, 2019. Recruitment started on September 18th, 2018 and is expected to be completed in March 2020.

## Data Availability

The datasets generated and analyzed during the current study are not publicly available to protect participant privacy but are available from the corresponding author on reasonable request.

## Abbreviations

ADHD: Attention Deficit Hyperactivity Disorder
CRI: Child Development Inventory
CiC: Caring in Chaos
DKK: Danish Krone
CONSORT: Consolidated Standards of Reporting Trials
EUR: Euro
FCD: Family Club Denmark
FDF: Voluntary Boy and Girl Association (Frivilligt Drenge- og Pige-Forbund)
GSE: General self-efficacy scale
HBSC: Health Behaviour in School-aged Children, a World Health Organization Collaborative Cross-national Study
ITT: Intention-to-treat
KFUM: Young Men’s Christian Association (Kristelig Forening for Unge Mænd)
PBI: Parent Behavior Inventory
PSS: The Parenting Stress Scale
RCT: Randomized Controlled Trial
SMD: Standardized mean difference
SWEMWBS: Short Warwick-Edinburgh Mental Well-being Scale
SPIRIT: Standard Protocol Items: Recommendations for Interventional Trials
